# The Effects of Multi-Theory Model–Based Behavior Change Intervention with Staircase Approach on Sedentary Lifestyle Among Community-Dwelling Older Adults: Study Protocol for a Randomized Controlled Trial

**DOI:** 10.2196/81284

**Published:** 2026-01-06

**Authors:** Subinuer Tuerdi, HuiLing Yang, Li He, Rong Yan, YaoYi Cui, XingHui Wang, ShanShan Dong, JiaYu Yang, KeQiang Zhang, Feng Li, YueWei Li

**Affiliations:** 1 School of Nursing, Jilin University, 965 Xinjiang St, Changchun, 130000, China, +86 13578873345; 2 Operating Room, Second Affiliated Hospital of Jilin University, Changchun, Jilin Province, China; 3 Nursing Department, The First Hospital of Hebei Medical University, Hebei, China; 4 Hepatopancreatobiliary Surgery Department, General External Center, First Hospital of Jilin University, Changchun, Jilin Province, China; 5 Medical Department, Second Hospital of Jilin University, Changchun, Jilin Province, China

**Keywords:** sedentary behavior, sedentary lifestyle, physical activity, older adults, behavior change, MTM, lifestyle intervention, multi-theory model

## Abstract

**Background:**

Sedentary lifestyles, as a nursing diagnosis, are prevalent in the life patterns of community-dwelling older adults, who have been shown to be the most sedentary and physically inactive subgroup. Prolonged low energy expenditure during waking hours leads to an increased risk of noncommunicable diseases and impairs physical functioning in older adults, negatively impacting their health outcomes. Therefore, interventions targeting changes in sedentary lifestyles are necessary to improve health behaviors in older adults.

**Objective:**

This study aimed to investigate the effect of the multi-theory model–based stepped behavior change intervention on sedentary lifestyle among community-dwelling older adults.

**Methods:**

This study is designed as a community-based, parallel-arm, assessor-blinded randomized controlled trial. Sixty participants were enrolled in this study and randomly assigned to the intervention group (received theory-based sedentary lifestyle change intervention) and the control group (received conventional behavioral change advice). End points were collected at baseline, immediately after the end of the intervention, week 12, and week 18. The primary endpoint is the change in self-reported sedentary time. Secondary endpoints include the changes in step count, time spent in light-intensity physical activity and moderate-to-vigorous-intensity physical activity, Measuring Change in Physical Activity Questionnaire score, Short Physical Performance Battery score, BMI, waist circumference, blood pressure, and Short-Form Health Survey-12-Version 2 score.

**Results:**

The study was initiated in May 2024. As of May 2025, the project had completed participant recruitment and data collection. The final manuscript with findings is expected to be submitted for publication in September 2025.

**Conclusions:**

This study uses a multi-theory model as its theoretical framework and adopts stepped sedentary lifestyle change as the intensity gradient of exercise behavior, creating a behavioral change pattern from sitting to standing and movement to light-intensity physical activity, to moderate-to-vigorous-intensity physical activity, and ultimately improving the sedentary lifestyle and obtaining health benefits, to provide community-dwelling older adults with individualized behavior change programs that are easy to adhere to and more applicable to daily activity patterns.

## Introduction

According to the United Nations World Population Prospects 2022 report, the average life expectancy at birth is expected to reach 77.2 years [1]. By the end of 2024, adults aged ≥65 years accounted for 15.6% of China’s total population [2]. However, increased longevity is often accompanied by more health problems and age-related diseases. As people age, their risk of disability and noncommunicable diseases rises, placing a heavy burden on both individuals and health care systems [3]. Sedentary behavior (SB) and physical inactivity (PI) are the two most significant public health issues among the modifiable factors. Preventing noncommunicable diseases and promoting healthy aging by addressing these issues are top priorities for organizations such as the World Health Organization [4-6].

According to the World Health Organization Guidelines on Physical Activity and Sedentary Behavior, older adults should restrict their sedentary time (ST), replacing ST with any intensity of physical activity (PA) provides health benefits. To mitigate the negative health effects of excessive SB, older adults should aim to do more than the suggested levels of moderate-to-vigorous-intensity physical activity (MVPA) [7]. Despite the well-known benefits of being physically active, SB and PI are still prevalent among older adults in China, and a large proportion of older adults do not meet the essential amount of PA required to maintain their wellness [8,9]. The North American Nursing Diagnostic Association has termed such behavioral patterns characterized by low–energy-expenditure waking-time activities as sedentary lifestyles and classified them as nursing diagnoses in the field of health promotion [10]. Both SB and PI can be considered as a sedentary lifestyle according to its defining features. Older adults are the most sedentary and physically inactive subpopulation [11,12].

Research shows that even if some seniors meet the recommended 150 minutes of MVPA per week (non-PI), they may still be sedentary, which can have a negative impact on metabolism and overall health [13]. SB is defined as any waking behavior characterized by an energy expenditure of 1.5 METs or lower while sitting, reclining, or lying [14]. Statistics show that the average ST of Chinese older adults is 9.6 hours per day, indicating that they spend a large proportion of their waking hours in SB [8]. Prolonged sitting time can result in decreased physical function, raised depression and anxiety, and an increased risk of chronic disease and all-cause mortality, all of which can have a significant impact on older adults’ health-related quality of life [15-21]. Therefore, effective interventions are needed to limit ST in older adults.

Common interventions targeting SB in community-dwelling older adults can be divided into two types: interventions that focus on reducing SB and approaches that target increased PA to reduce SB [22]. Research has shown that the former approach can lead to substantial reductions in ST [23]. Addressing prolonged SB independently of PA has many beneficial health outcomes, such as reduced risk of all-cause mortality and improved cardiometabolic risk factors [24]. However, these interventions may have several limitations. First, ST cannot be reduced endlessly. Findings from meta-analyses have suggested that total ST should be limited to less than 4 hours per day for those who are not physically active [25,26]. This goal may be difficult to achieve for the majority of older adults with chronic diseases, impaired cardiovascular health, or pain-related functional limitations [27]. Furthermore, sedentary habits in contexts such as reading and socializing should not be discouraged due to the mental health advantages and promotion of cognitive functioning in the aging process [28,29]. These limitations may also apply to other types of SB interventions. Second, the observed effects of SB reduction interventions in cardiometabolic biomarkers are generally inferior to those reported after exercise training interventions [30]. Interventions targeting the reduction of SB also include methods of interrupting ST—breaking and replacing ST with frequent brief periods of PA (ie, nonexercise daily living activities) to mitigate the adverse effects of prolonged SB [31]. Studies suggest that replacing ST with light-intensity physical activity (LPA) is associated with risk biomarkers such as waist circumference, high-density lipoprotein cholesterol, triglycerides, and insulin [32-34]. Where clinically meaningful improvements in these biomarkers occur, frequent and long-term interventions or a combination of PA interventions that replace SB with higher-intensity PA are needed [23].

For the second approach, the isotemporal substitution model posits that increasing the duration of one behavior leads to a decrease in the duration of one or more of the remaining behaviors. Therefore, it is feasible to increase an individual’s PA duration to decrease their SB [35]. Robust findings still suggest that sedentary individuals may benefit from PA interventions [24,36]. High sitting time is significantly associated with an increased risk of all-cause mortality and cardiovascular disease, which is especially pronounced in physically inactive individuals (those who do not meet the recommended minimum level of >150 min MVPA/wk), and the increased risk of ST can be effectively attenuated by engaging in PA above the guideline-recommended level [26,36,37]. The second intervention, aimed at increasing PA, also has some limitations, with low adherence and insignificant effects on reducing SB [23,38]. First, to attenuate the harms of prolonged SB, 60 to 75 minutes per day of MVPA is required. However, when the majority of individuals do not meet the recommended guidelines (ie, 30 min/d of MVPA, 5 d/wk), an additional 30 to 45 minutes per day of MVPA is impractical, which affects adherence to the intervention [26,27]. Second, despite participating in regular PA, the adverse health impacts of prolonged ST can diminish the benefits of physical exercise [39,40]. In conclusion, focusing only on one of the two interventions may not produce the desired results. Thus, the optimal prescription for changing a sedentary lifestyle should be based on the interaction between SB and PA and to find a balance between the w elements to better promote a healthy lifestyle [25].

**Figure 1.**
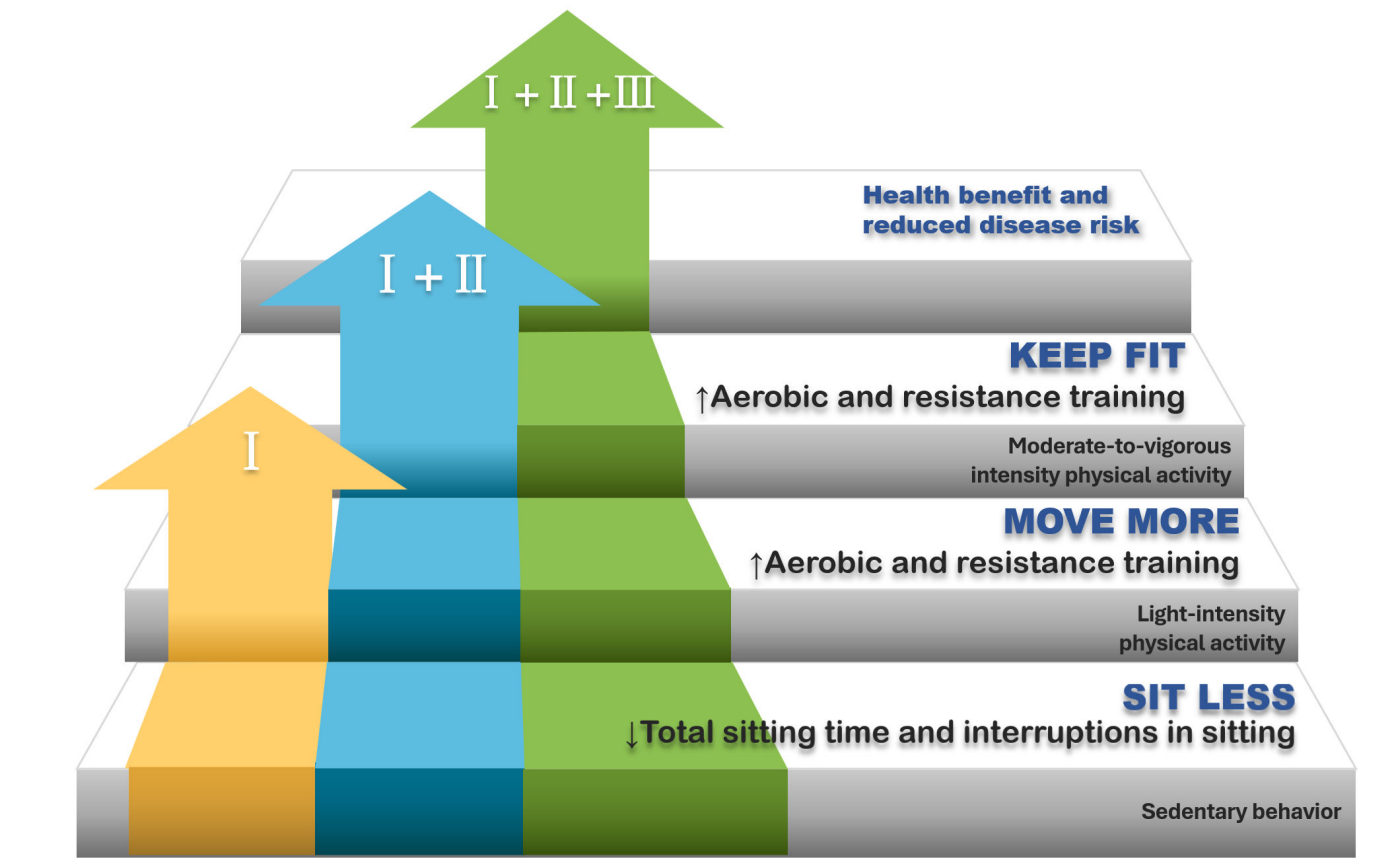
The “sit less, move more” strategy is based on the staircase approach.

Dunstan et al [25] in their study “Sitting Less and Moving More” suggest that sedentary lifestyle can be addressed through a “staircase approach” (Figure 1). This is a promising but understudied approach. It involves modest transition steps: first emphasizing the reduction of SB, decreasing overall sitting time, and breaking up ST by increasing standing and activity; then gradually increasing LPA to provide a “preparation base” for the long-term transition to higher-intensity PA, for example, walking, engaging in family leisure activities, flexibility training, strength training, etc; finally, gradually engaging in sufficient MVPA, such as brisk walking or jogging, dancing, tai chi, handball, water sports, etc, to meet the amount of PA above the guideline level [41]. Compared to accumulating weekly amounts of MVPA, interventions of the beginning stage aimed at reducing SB may seem more appealing and be easier for older adults to integrate into their daily lives [42]. Reducing SB may be an essential initial step toward implementing sustainable changes in movement patterns, which would support higher levels of overall PA and thus be beneficial to health.

As older adults have more time to establish links between contextual cues and SB and because most of them engage in SB without being aware of it, they are considered to have the most potential to develop strong sedentary habits [43,44]. Therefore, breaking habitual SB and sustaining health behavior change over time is considered one of the greatest obstacles to this intervention. The multi-theory model (MTM) of health behavior change can be used to explain and predict the initiation and maintenance of health behavior change and can be applied at the individual, population, and community levels [45]. It consists of 2 components: the initiation and the maintenance of health behavior change. The initiation of health behavior change is the process of transitioning from one behavior to another, which means a one-time behavior change. It includes 3 components: participatory dialogue (interactive communication about the pros and cons of health behavior change), behavioral confidence (a way to gauge a person’s level of certainty that they will participate in changing a health behavior in the future), and changes in physical environment (the environment in which the behavior is enacted). The maintenance of health behavior change is the same as the long-term performance of the health behavior change. It involves emotional transformation (which refers to overcoming self-doubt, inertia, and impulsivity, focusing one’s feelings and emotions on health behavior change, directing oneself moving toward a goal), practice for change (which emphasizes reflective behavior, including continuously and prudently considering behavior change, combined with the ongoing correction to remove ineffective strategies and address barriers), and changes in social environment (building social support in the environment, using positive relationships to achieve desired behaviors). As a comprehensive theoretical model, MTM has been successfully applied in the initiation and maintenance of health behavior changes, such as PA, vaccination, and smoking and alcohol cessation [46-48].

Behavior change techniques (BCTs) are an observable, replicable, and nonreducible component of an intervention [49]. To evaluate interventions based on the MTM, multiple BCTs were selected to map onto the structure of the theoretical model. This study seeks to better describe and make transparent the intervention by combining the theoretical structure with the behavioral change techniques.

Using the theoretical framework of the MTM and the corresponding BCTs, a sedentary lifestyle intervention program for older adults based on the “staircase approach” was constructed. An experimental study will be conducted to verify whether a sedentary lifestyle modification intervention based on the MTM and the staircase approach is more effective than conventional education in reducing ST and improving PA levels, physical function, anthropometric parameters, blood pressure, and quality of life among community-dwelling older adults.

## Methods

### Study Design

The study protocol adhered to the SPIRIT guidelines (Checklist 1) [50]. The Ethics Committee of the School of Nursing, Jilin University, approved the ethical clearance for the project (2023122001). The trial was registered in the China Clinical Trial Registry on March 25, 2024 (ChiCTR2400082225). This is a multicenter, assessor-blinded, 18-week, two-arm, parallel-group, randomized controlled trial examining the effects of an MTM-based sedentary lifestyle change intervention for older adults on SB, PA, and health outcomes compared with usual education.

### Participants

Participants for this study were recruited through posters and leaflets distributed at 2 community health service centers in Changchun City, Jilin Province, China, from March 2024 to October 2024. All participants signed informed consent before the baseline outcome assessment. The following are the inclusion and exclusion criteria for participants:

Inclusion criteria:

1. Older adults aged ≥65 years.
2. Residents living in the community for ≥6 months.
3. Sedentary lifestyle (ie, more than 6 h of time awake spent in a sitting or reclining posture per day and PI: MVPA ≦150 min/wk) for at least 6 months.
4. Able to walk 100 m without assistance.
5. Informed consent and voluntary participation in this study.

Exclusion criteria:

1. Severe physical illness or long-term bedridden.
2. Unable or unwilling to communicate with researchers.
3. Participating in other PA or SB intervention programs.
4. Severe cardiovascular and cerebral vascular diseases that require restriction of activities.
5. Severe muscle weakness, skeletal diseases, or deformities that lead to restriction of PA.
6. Depression, schizophrenia, and other psychiatric disorders or dementia that led to severe cognitive impairment.
7. History of central nervous dysfunction, such as mild hemiplegia, spinal cord disease, and cerebral ataxia.

### Study Interventions

Using the MTM as a framework and based on the review of relevant guidelines and literature, the sedentary lifestyle intervention program for community-dwelling older adults was developed (Table 1). Multimedia Appendix 1 shows the mapping of the sedentary lifestyle change interventions in the MTM, and Multimedia Appendix 2 shows the mapping of the sedentary lifestyle change interventions in the “staircase approach.” Participants in the intervention group will receive a sedentary lifestyle modification intervention based on the MTM and the staircase approach. Participants in the usual care group will receive a group education session (non-stepwise) on changing sedentary lifestyles and will be given a healthy behavior manual, an activity diary, and a pedometer.

**Table 1.** The multi-theory model (MTM)–based sedentary lifestyle intervention program for community-dwelling older adults.

Timing and interventions	MTM constructs	BCTs^a^	Content of interventions	Duration/frequency
Week 1‐2, Phase I				
Discussion of advantages and disadvantages	Participatory dialogue/emotional transformation	Pros and cons	In small groups of 2‐3 people, participants engage in face-to-face communication with health educators to discuss the advantages and disadvantages of sedentary lifestyles and to identify the main factors contributing to sedentary lifestyles, concerns regarding behavior change and the home environment in order to provide personalized advice.	20 min/group
Thematic session on sedentary behavior change	Behavioral confidence	Information about health consequences Credible sources Instruction on how to perform the behavior Problem solving	This session will focus on the recognition and prevalence of SB^b^, the harms of being sedentary, the benefits of reducing sitting time, etc Peer sharing: sharing the adverse effects of long-term SB through the experiences of others, video cases, etc Recommendations for reducing SB—interrupting or replacing SB with standing or LPA^c^, household domain physical activity in different settings where it occurs, etc Give personalized guidance on the main causes of SB for each older adult. Co-discuss the barriers that participants may encounter during the process of SB change and provide practical solutions (after discussion of advantages and disadvantages).	60 min
Healthy Behavior Manual	Behavioral confidence/changes in physical environment	Information about health consequences Demonstration of the behavior Instruction on how to perform the behavior	The manual contains the same information as presented in the two sessions: Common patterns of SB, their negative effects, the advantages of reducing SB, and advice on how to break prolonged sitting. The benefits of engaging in regular PA^d^, tips on how to do it, such as aerobic, resistance, balance, and flexibility training, as well as the related graphic explanations.	One for each person
Sedentary behavior interruption intervention	Changes in physical environment/practice for change	Prompts/cues Behavior substitution	Participants will receive the Cube Timer with a 5-15-25-45 min preset countdown function. It is a simple, easy-to-carry, and button-free time reminder tool. The researchers recommend that participants anticipate the duration of occurrences of SBs before they proceed with them and turn the selected time face up to start timing (choose less than 25 min if possible). When the time is up, the timer emits a continuous beep, indicating that the participant is getting up and moving around, and then the cycle of timekeeping is repeated, thus interrupting the SB. Encourage participants to set at least one if-then planning for themselves and stick to it over the next few weeks, for example, stand when speaking on the phone, put the remote control in the distance when watching TV^e^, get off the bus one stop earlier, stand during TV adverts, drink plenty of water to promote urination (if applicable), etc	Breaks the sitting time every 5/15/25/45 min
Week 3‐4, Phase II				
Warning stickers	Changes in physical environment	Prompts/cues	The stickers were predesigned by the research team, including short and eye-catching sentences, and their content was developed based on motivational phrases and health-related proverbs that resonate well with older adults in China, aiming to effectively raise awareness of sedentary risks and encourage behavior change. Participants can post them where they regularly experience SB. Examples of the sticker messages include: “Flowing water never stagnates, a moving body stays strong,” “Walk a hundred steps after meals, live to ninety-nine,” “Feeling stuck to the chair? Stand up, take a walk, and break up with it!,” “Start your day with Baduanjin, end it with Tai Chi grace.”	Three stickers per person
Pedometer-based interventions	Changes in physical environment/emotional transformation/practice for change	Self-monitoring of behavior Graded tasks Material reward	Pedometers will be used for self-monitoring of PA and to record step counts. The intervention consisted of the following: Educating participants on the benefits of walking and the necessity of maintaining a certain number of steps every day, Participants will be instructed to record their daily step count in an activities diary, create a desired step goal, and review the objective to detect any gaps and regulate their activity level. The step goal is personalized to the participant. For older adults with less than 5000 steps at baseline, it is advised to increase the objective by at least 500 steps each week until it is maintained at more than 3000 steps from baseline. Participants with high completion of the activity diary and who achieved and maintained the weekly target ST^f^, step count, and PA were materially rewarded at the end of the intervention and at the end of the follow-up period.	One for each person
Activities diary	Emotional transformation/practice for change	Self-monitoring of behavior Action planning Review behavior goals Goal setting	Participants can set weekly goals for sedentary time (min/day), step count (steps/day), and MVPA^g^ time (min/week) in the goal-setting table of the diary. Goals can be updated every 7 days. Participants can record their daily sitting time (total time, the longest continuous sitting time, prolonged sedentary bouts) in the corresponding context of the SB recording form. The diary includes a free recording section, where participants can record daily action plans, problems encountered, and experiences gained during the process of behavioral change. The activity diary is also used to review the participant’s behavioral goals, overcome self-inertia, focus on the individual’s emotions on healthy behavior change, and direct them toward their goals. The activities diary emphasizes reflective behavior, continual critical consideration of behavior change, and removal of ineffective strategies.	One for each person
Week 5‐6, Phase III				
Thematic sessions on physical activity	Behavioral confidence	Information about health consequences Demonstration of the behavior Instruction on how to perform the behavior Graded tasks	This session highlights the following: The significance of PA, the risks associated with PI^h^, and the advantages of physical exercise for health Forms of exercise, time, control of the intensity, conditions in which exercise should not be performed, warming up and stretching safely The main points of exercise will be presented through text, diagrams, videos, and demonstrations, following the guidelines of the WHO^i^ on physical activity and sedentary behavior and the expert consensus of the International Conference on Frailty and Sarcopenia Research. Through a combination of structured and integrated lifestyle activities, multicomponent exercise forms are recommended, including aerobic, plyometric, balance, and flexibility training (VIVIFRAIL). To achieve optimal levels of adherence, it is recommended to start with a single form of exercise and allow older adults to gradually adapt to the new form of exercise before adding other components.	90 min
Introduction to health and fitness resources	Changes in physical environment	Reconstructing the physical environment	To improve recognition and utilization of health resources, participants will be introduced to local gym facilities, medical resources, and recreational facilities (stadiums, badminton courts, table tennis tables, etc) available in the neighborhood.	30 min
Social support	Changes in social environment	Social reward Reconstructing the physical environment	Contacting the patient’s family members or partners and seeking their supervision and cooperation so that the patient can receive their encouragement and support. Call on community health service providers to join in the support of sedentary lifestyle change for the participants. Publicize the benefits of sedentary lifestyle change by posters in order to dispel the community’s concerns about PA in older adults.	20 min
Week 7‐18				
Telephone follow-up	Changes in social environment	Problem solving	During the follow-up period, two telephone consultations will be conducted to gather information on the implementation of behavioral change, provide advice to participants on how to overcome barriers to behavioral change, and encourage the continuation of good lifestyle habits.	20 min

a BCT: behavior change technique.

b SB: sedentary behavior.

c LPA: light-intensity physical activity.

d PA: physical activity.

e TV: television.

f ST: sedentary time.

g MVPA: moderate-to-vigorous-intensity physical activity.

h PI: physical inactivity.

i WHO: World Health Organization.

The program for the intervention group will follow the staircase approach in the rhythm of the reduction of ST and interruption of SB through standing and moving, followed by gradual replacement of SB with LPA, and ultimately leading to increased PA and modification of a sedentary lifestyle. During weeks 1‐2, “Phase I: Sitting Less” will be implemented. This phase includes a discussion of advantages and disadvantages, thematic session on SB change, Healthy Behavior Manual, and SB interruption interventions. This component involves recognition of SB and reduction and interruption of SB through standing and activities. “Phase II: Moving More” will be implemented during weeks 3‐4. The interventions will include warning stickers, pedometer-based interventions, and activities diary. This component involves the gradual replacement of SB with LPA. “Phase III: Increasing Physical Activity and Modifying Sedentary Lifestyle” will be implemented during weeks 5‐6. The interventions will include thematic sessions on PA, introduction to health and fitness resources, and social support. This intervention aims to increase and maintain the amount of MVPA among participants. They will be encouraged to engage in all stages of the intervention, and data will also be incorporated if the participants stay at any stage of the staircase lifestyle change intervention, provided that they have attended phase I (weeks 1‐2); otherwise, they are deemed to have withdrawn.

### Study Measurements

#### End Points

**Figure 2.**
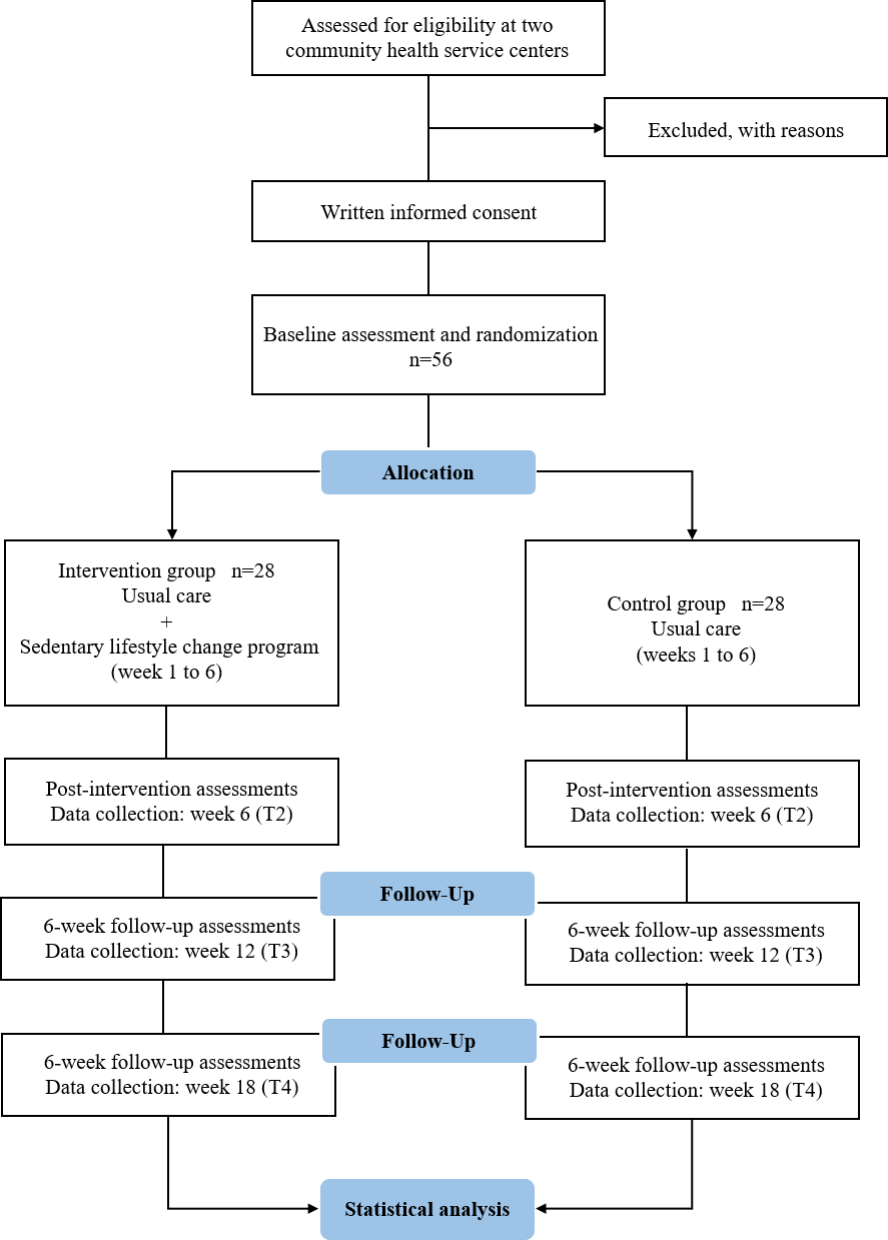
CONSORT flow diagram.

The duration of the intervention is 6 weeks with 12 weeks of follow-up. Outcome measures were collected at baseline T1, end of intervention (week 6) T2, week 12 (T3), and week 18 (T4; Figure 2). The primary endpoint is the change in self-reported ST (total ST and SB characteristics, such as screen-based ST, longest continuous ST, and number of sedentary bouts ≤30 min). Secondary endpoints include the changes in step count (steps/d), time spent in LPA (min/wk), time spent in MVPA (min/wk), the Short Physical Performance Battery (SPPB) score, blood pressure, Measuring Change in Physical Activity Questionnaire (MCPAQ) score, waist circumference, BMI, and quality of life (SF-12v2 score) (Table 2).

**Table 2.** Participant schedule of enrollment, interventions, and assessments.

	Study period				
	Enrollment	Baseline assessment/allocation	Post allocation		
Timepoint	T0	T1	T2 6 wk	T3 12 wk	T4 18 wk
Eligibility screen	×				
Informed consent	×				
Allocation		×			
Interventions					
CON group		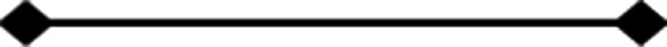			
INT group		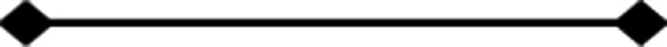			
Assessments					
Sociodemographics		×			
Primary outcomes					
Sedentary time (total sedentary time and sedentary behavior characteristics)		×	×	×	×
Secondary outcomes					
Step count (Omron HJ-204)		×	×	×	×
Time spent in LPA/MVPA (IPAQ-S-C)		×	×	×	×
Measuring Change in Physical Activity questionnaire (MCPAQ)		×	×	×	×
Physical function (SPPB)		×	×	×	×
Quality of life (SF-12)		×	×	×	×
BMI		×	×	×	×
Waist circumference		×	×	×	×
Blood pressure		×	×	×	×

a SF-12: Short-Form Health Survey-12.

#### Measurements

A self-developed questionnaire will be used to investigate the sociodemographic and clinical information of the participants, including age, gender, ethnicity, educational level, marital status, illness, residence, risk of falls, frailty status, current employment status, and availability of exercise areas in the vicinity of the residence.

##### ST and PA

The International Physical Activity Questionnaire Short version Chinese (IPAQ-S-C) was used to assess participants’ levels of PA and SB over the past 7 days. It consists of vigorous intensity, moderate intensity, walking (light intensity), and ST, with a total of 7 items. Qu et al [51] conducted a cross-cultural adaptation to form a Chinese version of the IPAQ. The reliability and validity of the Chinese version of the questionnaire were favorable. Total ST was recorded as the average amount of time participants spent in sitting, lying, and semi-lying postures each waking hour. When participants have difficulty recalling total ST, recall will be aided by possible sedentary scenarios in a “typical day,” such as calculating the total amount of ST spent daily in transportation, hobbies, watching TV, smartphones and computers, socializing, reading, eating, and any other SBs.

SB characteristics, including screen-based ST, longest continuous ST, and number of sedentary bouts lasting ≤30 minutes, were obtained through a self-developed questionnaire. Screen-based ST was recorded as ST occurring in situations such as watching television and using mobile phones, computers, and other similar electronic devices.

##### Step Count

Participants’ PA will be monitored by using Omron HJ-204 pedometers, which collect the average number of steps taken over 7 days, in steps per day. Participants will be asked to wear the pedometer over their hip and to record the number of steps in the activities diary before going to bed.

##### Measuring Change in PA

The MCPAQ was developed by Nahar et al [52] based on an MTM with 29 items containing 2 subscales, initiation and maintenance. The questionnaire is based on a 5-point Likert scale, with values ranging from 0 (never) to 4 (completely sure). The initiation model consists of 3 constructs: participatory dialogue (10 items), behavioral confidence (5 items), and changes in physical environment (3 items). The maintenance model consists of emotional transformation (3 items), practice for change (3 items), and changes in social environment (3 items). The higher the total score for each item, the greater the likelihood that participants will initiate or maintain changes in PA. Yang et al [53] conducted a cross-cultural adaptation to develop the Chinese version of the MCPAQ. The Chinese version of the MCPAQ has shown good reliability and validity in Chinese older adults with hypertension. The total scale Cronbach *α*=0.911, the subscales ranged from 0.729 to 0.943, and the test-retest reliability was 0.898.

##### Physical Function

SPPB will measure participants’ physical function [54], which combines balance, gait speed, and leg strength into a single score from 0 (worst) to 12 (best). Meaningful clinical change is considered 1 point for the SPPB [55]. A total score of <10 is considered frailty, as well as a high risk of disability and falls. For increased PA, participants were categorized based on SPPB scores at baseline to recommend a personalized Vivifrail multicomponent exercise program at phase III [56].

##### Quality of Life

Participants’ quality of life will be assessed with the Chinese version of the Short-Form Health Survey-12-Version 2 (SF-12v2), one of the common measurement tools for evaluating health-related quality of life from the patient’s perspective [57]. The scale has demonstrated high reliability and validity in evaluating the health status of Chinese older adults [58,59]. The scale consists of 12 items, including 8 dimensions, which are physical functioning (2 items), role physical (2 items), bodily pain (1 item), general health (1 item), vitality (1 item), social functioning (1 item), role emotional (2 items), and mental health (2 items). The first 4 dimensions are within the physical health composite scale, and the rest are part of the mental health composite scale. It is recommended to use the total scores of the physical and mental composite scale, with a mean score of 50 (SD 10).

##### Anthropometric Parameters

Anthropometric parameters included height, weight, waist circumference, BMI, and blood pressure. Each data collection will be repeated twice using the same measuring tool, and the average value recorded will be rounded to one decimal.

##### Blood Pressure

Avoid strenuous exercise or workout within 1 hour before measurement. The participants will sit still for 5 to 10 minutes; the mercury sphygmomanometer is used to measure the systolic and diastolic blood pressure.

##### Adherence

The weekly sessions and interventions will be repeated 2 to 4 times in the community health center to avoid scheduling conflicts for participants and to ensure compliance. The percentage of participants who attend sessions and activities will be recorded (total number of activities/actual number of sessions attended). Participants’ satisfaction with the intervention will be assessed by rating the study on a scale of 1 to 10.

##### Adverse Events

Adverse events will be assessed through participant self-reporting. The number of falls, hospitalizations in the last 5 months, and emergency room visits will be recorded for participants during the study.

### Sample Size Calculation

The sample size was calculated using G*Power software (version 3.1.9.6) [60]. The sample size estimation refers to the standardized deviation of the self-reported ST among community-dwelling older adults obtained from a similar previous study [42]. A total of 48 older adults (24 per group) will be required to detect the effect size of 0.84, with a power of 80% (1−*β*=0.80) and a level of significance of 5% (*α*=.05, 2-tailed) by unpaired *t*-test. Using an SD of 142.98 minutes per day and an expected reduction of 120 minutes per day self-reported sitting time, a sample size of 24 patients per arm is needed. Taking a dropout of 15% into consideration, we will have at least 56 participants in our study.

### Randomization and Blinding

Participants will be randomized in a 1:1 ratio into a behavioral intervention group, which receives a staircase MTM-based SB change intervention and a usual care group, which receives recommendations for reducing SBs. Randomization will be stratified by center, and each center will be grouped by age 65 to 75 and ≥75 years (as older adults aged ≥75 y are more likely to experience mobility impairment due to chronic conditions, resulting in higher ST compared to those aged 65‐75 y) [19,61]. Using a permuted block randomization, with a block size of 4, allocation sequences will be generated online using a website (www.randomizer.org) by an independent professional who will not be involved in subject recruitment, intervention implementation, or outcome assessment for this study, and investigators will be informed of group assignment by telephone.

### Investigators

The research team consisted of nursing specialists, community nurses, rehabilitation therapists, and postgraduate nursing students. Before implementing the intervention, all staff received comprehensive training from the principal investigator and participated in simulation training to grasp the important elements of the intervention and effectively implement it.

### Dropout and Withdrawal

Participants can withdraw at any time for any reason. They can also choose to remove their data from the study. Participants will be regarded as having withdrawn if they request to leave the trial or are lost to follow-up. Participants in the intervention group will be regarded as having withdrawn if they no longer participate in the behavior change intervention that differed from those in the control group. As this research assumes that each stage of the staircase intervention generates health effects, participants will be allowed to skip a stage but will be expected to complete the first stage and be considered to have withdrawn if they do not.

### Statistical Analyses

Data analyses follow intention-to-treat principles, using generalized estimating equation models to assess differential changes in the outcome variables between the 2 groups from baseline to 12 weeks (group×time interaction), with adjustment for potential covariates as appropriate (ie, baseline group differences at 2-sided *P*<.25). Effect sizes for continuous outcome variables were estimated using Cohen *d* statistic based on between-group mean differences from baseline T1 to T2, T3, and T4 end points with cutoffs set at 0.2 (small), 0.5 (medium), and 0.8 (large) [62]. Each generalized estimating equation model includes the main effects of group (intervention vs control), time (T2, T3, T4 vs baseline), and 2-way interaction effects (group×time). The group difference in the change from baseline to 6, 12, or 18 weeks between the 2 groups is verified when the 2-way interaction effects (group×time) was statistically significant. All statistical analyses are 2-sided and performed using IBM SPSS 25.0 software. *P* values <.05 is considered statistically significant.

Continuous variables that conform to normal distribution will be expressed as mean (SD) using an independent samples *t* test. Continuous variables that are not normally distributed and ranked variables will use the Mann-Whitney *U* test, expressed as median (M) and quartiles (P₂₅-P₇₅). Categorical table variables will be statistically described by frequency and percentage (%) using *χ*² test or Fisher exact test.

### Adverse Events and Audit

The research team will collect, report, and assess adverse events to determine their severity and whether they are related to this trial. Serious adverse events and reactions will be reported to the Ethics Committee and appropriate measures will be taken (if present). Audits will be conducted by the School of Nursing, Jilin University.

### Data Collection, Monitoring, Management, and Storage

The principal investigator will input the collected data into a computerized database. Participants’ names will be anonymized and replaced with codes. All other study data will be securely stored on encrypted computer drives in the Research Centre of Jilin University School of Nursing. Only the research team will have access to the data. Data will be securely archived at the end of the study and stored for up to 3 years.

### Ethical Considerations

The design and procedures of this controlled trial followed the Declaration of Helsinki. The study obtained approval from the Ethics Committee of the School of Nursing, Jilin University on December 20, 2023 (grant 2023122001). Written informed consent will be obtained from each participant before baseline measurements. After the follow-up data collection was completed and in accordance with their expressed preferences and needs, participants in the control group were provided with the intervention components and material support they had missed. The study’s implementation adheres strictly to the principles of beneficence, respect for human dignity, justice, and respect for truth and fact.

## Results

The study was initiated in May 2024. As of May 2025, the project had completed participant recruitment (60 participants) and data collection. The final manuscript with findings is expected to be submitted for publication in September 2025. This protocol is published to ensure methodological transparency and to invite peer review focused on study design.

## Discussion

### Anticipated Findings

We hypothesize that community-dwelling older adults receiving the MTM-based stepped intervention for sedentary lifestyle change will demonstrate significantly greater improvements across multiple outcomes compared to those in the conventional health education group. Specifically, the intervention group is anticipated to exhibit a pronounced reduction in total ST, longest continuous ST, and screen-based ST. Concurrently, we expect increases in daily step counts, frequency of sedentary breaks, and time spent in MVPA. These behavioral changes are further projected to translate into meaningful health benefits, including improved quality of life, as well as modest reductions in blood pressure, BMI, and waist circumference.

### Comparison to Previous Work

The evidence indicates that older adults spend approximately 65% to 80% of their waking hours sedentary [63]. In a survey of Chinese older adults, 26.1% participated in regular PA (more than 3 times a week, each time for more than 30 min of MVPA) [64], suggesting that sedentary lifestyles may be prevalent among older adults. The long-term presence of low energy expenditure during waking hours is detrimental to physical health, mental health, and quality of life and exacerbates the adverse outcomes [36]. Therefore, interventions targeting changes in sedentary lifestyles are necessary to improve health outcomes in older adults. Previous research has confirmed the separate benefits of reducing SB and increasing PA. However, the feasibility and efficacy of a comprehensive, stepped intervention that systematically integrates both strategies to modify sedentary lifestyles in older adults have not been adequately examined.

Most current interventions for PI in older adults involve the traditional “elevator approach,” that is, interventions aimed directly at increasing PA, and tend to ignore the potential SB of the target population [41]. Despite increased PA, older adults may still engage in SB [65,66]. As the health effects of SB are independent of PA, too much cumulative SB can still harm overall health and make it difficult to produce the desired health benefits [67,68]. Similarly, modifying SB alone—without emphasizing the quantity of MVPA—the presence of PI still leads to an inability to efficiently achieve the desired health benefits [36]. Our study addresses this critical gap by proposing an integrated, staircase approach-based sedentary lifestyle intervention that simultaneously targets SB reduction and PA promotion, guided by the MTM.

The MTM suggests that for initiation of behavior change, individuals perceive that the advantages of behavior change outweigh the disadvantages, and they have behavioral self-confidence and are supported by the physical environment. To maintain behavior change, it is necessary to translate one’s feelings into goals, to constantly strive for change, and to have the support of the social environment [45]. Rather than simply applying behavioral change theory, our approach was informed by the specific characteristics of the older adults (eg, cognition, environment, social norms), leading to a targeted selection of components and design of strategies. According to qualitative studies, older adults are often unaware of the benefits of reducing SB, resulting in low self-efficacy [69,70]. Therefore, there is a need to provide knowledge-based sessions on SB for older adults, emphasizing its advantages and disadvantages and the importance of behavioral change and offering practical tools [43]. For instance, the cube timer was selected as a tool to interrupt SB. Interventions aimed at breaking up ST through standing and LPA throughout the day may be an effective and more feasible option for improving health [42,71]. This initial step within the staircase approach laid the foundation for subsequently introducing higher-intensity PA, thereby demonstrating a systematic implementation that transcends previously fragmented applications. Higher daily step counts are associated with higher LPA or SB ratios and greater time spent on MVPA [72]. This study also added a pedometer-based intervention that included sessions on the benefits of walking and recommendations for self-monitoring daily steps with a pedometer. Pedometer and cube timer serve as low-technological-threshold, cost-efficient tools that may present a pragmatic alternative to many digital interventions by overcoming the technological usage barriers commonly encountered by older adults [43].

Distinct from prior research predominantly focused on individual-level behavior change, this study also specifically focuses on the impact of sociocultural environments on SB in older adults. Consequently, our intervention design incorporates components aimed at mobilizing social support to address this documented, yet frequently overlooked challenge in existing interventions. Existing sociocultural expectations and social norms are supportive of older adults’ seated position, considering it “safe” for them, and supporting their inactivity as a “benevolent” act or, in some settings, standing up and moving around when everyone is sitting is considered “embarrassing” behavior [28,69,73]. These viewpoints may hinder older adults’ capacity to change their SB in community settings. Therefore, our intervention integrates strategies to engage participants’ close social networks and community members, creating a supportive environment that aligns with the changes in the social environment construct of the MTM.

### Strengths and Limitations

This research has several strengths. First, our study takes community-dwelling older adults as the research population; applying a sedentary lifestyle modification intervention program based on the MTM model, and through the chosen behavior-change techniques, acting on the participants’ behavior, physical and social environments, addresses the problems of difficulty in breaking habitual SB and low adherence to behavioral modification and ultimately achieves the gradual transformation of SB into PA of different intensities and long-term maintenance of behavioral change. Second, this study uses the “staircase approach” as an activity intensity gradient for modifying sedentary lifestyles. It facilitates a transition from SBs to standing and light activities, then to LPA, and further to MVPA, ultimately establishing a sustainable behavior change model that delivers health benefits. The program provides community-dwelling older adults with sedentary lifestyles a personalized behavior change program that is easier to adhere to and better suited to their daily activity patterns.

There are also several limitations. The modest sample size in this study may constrain the generalizability and statistical power of the outcomes. Moreover, the reliance on self-reported measures for SB introduces potential recall bias. Future investigations should utilize objective monitoring devices, such as accelerometers, to enhance data accuracy and adopt multicenter designs with larger cohorts to validate the external validity of the findings. Extending both the intervention and follow-up durations is also recommended to better evaluate the long-term sustainability of the intervention effects.

### Future Directions

This study offers valuable practical implications for nursing practice in health management of community-dwelling older adults. For older adults with sedentary lifestyles, the MTM-based stepped intervention demonstrates potential in systematically reducing SB and promoting MVPA through a progressive intensity gradient, thereby facilitating health benefits. It is recommended that community health centers incorporating such interventions into routine health management programs, promoting the use of low-cost prompting tools (eg, cube timer), combined with personalized goal-setting and social support, is essential to enhance participant engagement and adherence. Furthermore, future initiatives may explore the integration of digital tools (eg, mobile app reminders) with the stepped intervention model to improve both the accessibility and scalability of the intervention.

### Dissemination

Participants will be informed of the results of the study through a summary report. Study results will be disseminated through journal publications. The anonymized participant dataset will be retained in an appropriate data archive for 3 years after the study’s end for sharing. Without participant approval, the results of this study and any information submitted by participants will not be disclosed to any third party.

## Supplementary material

10.2196/81284 Multimedia Appendix 1 The mapping of the sedentary lifestyle change interventions in the multi-theory model.

10.2196/81284 Multimedia Appendix 2 The mapping of the sedentary lifestyle change interventions in the “staircase approach.”

10.2196/81284 Checklist 1 SPIRIT 2013 checklist.

## Abbreviations

BCTs: behavior change techniques
IPAQ: International Physical Activity Questionnaire
LPA: light-intensity physical activity
MCPAQ: Measuring Change in Physical Activity Questionnaire
MTM: multi-theory model
MVPA: moderate-to-vigorous-intensity physical activity
PA: physical activity
PI: physical inactivity
SB: sedentary behavior
SF-12v2: The Short-Form Health Survey-12 version 2
SPPB: Short Physical Performance Battery
ST: sedentary time
