# Supplementary material for: The Effects of Multi-Theory Model–Based Behavior Change Intervention with Staircase Approach on Sedentary Lifestyle Among Community-Dwelling Older Adults: Study Protocol for a Randomized Controlled Trial
Source: JMIR Res Protoc. 2026 Jan 6;15:e81284. doi: 10.2196/81284 (PMC12772486; doi:10.2196/81284)
Supplement: Multimedia Appendix 1 [file resprot-v15-e81284-s001.docx]

**Multimedia Appendix 2** The mapping of the sedentary lifestyle change interventions in the MTM

| Behavior change techniques | Interventions |  | Multi-theory model constructs | Behavioral objectives | Behavioral outcomes |
| --- | --- | --- | --- | --- | --- |
| Pros and cons | Discussion of advantages and disadvantages | 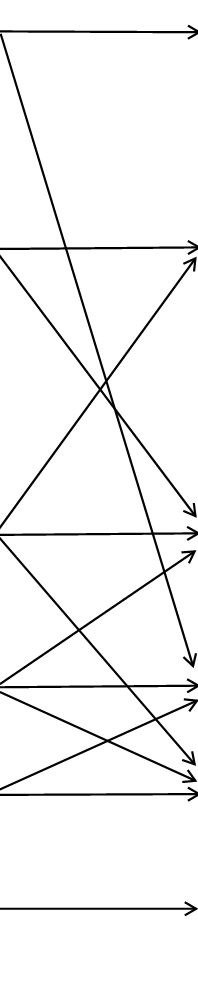 | **Participatory dialog** | **Initiation**  **/One-time behavior change** | **Sedentary lifestyle changes** |
| Information about health consequences, Credible sources,  Instruction on how to perform the behavior, Problem solving | Thematic session on sedentary behavior change |  | **Behavioral confidence** |  |  |
| Information about health consequences, Demonstration of the behavior, Instruction on how to perform the behavior | Healthy behavior manual |  |  |  |  |
| Information about health consequences, Demonstration of the behavior, Instruction on how to perform the behavior, Graded tasks | Thematic Sessions on Physical Activity |  |  |  |  |
| Prompts/Cues, Behavior substitution | Sedentary behavior interruption intervention |  | **Changes in physical environment** |  |  |
| Prompts/Cues | Warning sticker |  |  |  |  |
| Reconstructing the physical environment | Introduction to health and fitness resources |  |  |  |  |
| Self-monitoring of behavior, Graded tasks, Material reward | Pedometer-based interventions |  | **Emotional**  **transformation** | **Sustenance/Long-term behavior change** |  |
| Goal setting, Self-monitoring of behavior,  Action planning, Review behavior goals | Activities diary |  | **Practice for change** |  |  |
| Social reward  Reconstructing the physical environment | Social support |  | **Changes in social environment** |  |  |
| Problem solving | Telephone follow-up |  |  |  |  |
