# Supplementary material for: The Effects of Multi-Theory Model–Based Behavior Change Intervention with Staircase Approach on Sedentary Lifestyle Among Community-Dwelling Older Adults: Study Protocol for a Randomized Controlled Trial
Source: JMIR Res Protoc. 2026 Jan 6;15:e81284. doi: 10.2196/81284 (PMC12772486; doi:10.2196/81284)
Supplement: Multimedia Appendix 2 [file resprot-v15-e81284-s002.pdf]

Multimedia Appendix 2

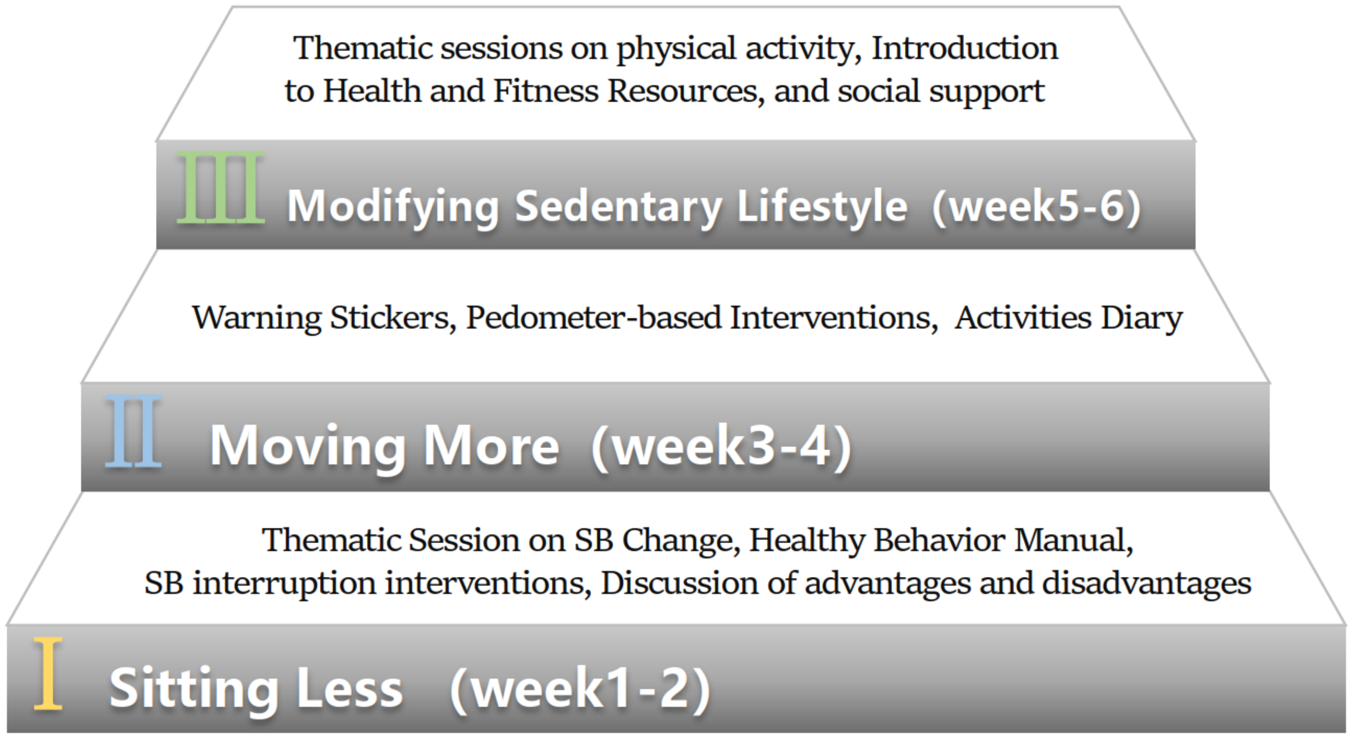

The mapping of the sedentary lifestyle change interventions in the “staircase approach”
